# FluoroSpot Analysis of TLR-Activated Monocytes Reveals Several Distinct Cytokine-Secreting Subpopulations

**DOI:** 10.1111/j.1365-3083.2011.02641.x

**Published:** 2012-02

**Authors:** C Smedman, T Ernemar, L Gudmundsdotter, P Gille-Johnson, A Somell, K Nihlmark, B Gårdlund, J Andersson, S Paulie

**Affiliations:** 1 Division of Infectious Diseases, Department of MedicineKarolinska Institute, Stockholm, Sweden; 2 Mabtech ABNacka Strand, Sweden; 3 Department of Clinical Science, Intervention and Technology, Karolinska InstituteStockholm, Sweden

## Abstract

Monocytes have long been considered a heterogeneous group of cells both in terms of morphology and function. In humans, three distinct subsets have been described based on their differential expression of the cell surface markers CD14 and CD16. However, the relationship between these subsets and the production of cytokines has for the most part been based on ELISA measurements, making it difficult to draw conclusions as to their functional profile on the cellular level. In this study, we have investigated lipoteichoic acid (LTA)- and lipopolysaccharide (LPS)-induced cytokine secretion by monocytes using the FluoroSpot technique. This method measures the number of cytokine-secreting cells on the single-cell level and uses fluorescent detection, allowing for the simultaneous analysis of two cytokines from the same population of isolated cells. By this approach, human monocytes from healthy volunteers could be divided into several subgroups as IL-1β, IL-6, TNF-α and MIP-1β were secreted by larger populations of responding cells (25.9–39.2%) compared with the smaller populations of GM-CSF (9.1%), IL-10 (1.3%) and IL-12p40 (1.2%). Furthermore, when studying co-secretion in FluoroSpot, an intricate relationship between the monocytes secreting IL-1β and/or IL-6 and those secreting TNF-α, MIP-1β, GM-CSF, IL-10 and IL-12p40 was revealed. In this way, dissecting the secretion pattern of the monocytes in response to TLR-2 or TLR-4 stimulation, several subpopulations with distinct cytokine-secreting profiles could be identified.

## Introduction

Monocytes and macrophages are key cells of the innate immune system. Here, they fulfil a series of important functions relating to their phagocytic capacity, their role as antigen-presenting cells and their ability to produce and secrete a large number of signalling molecules including pro- and anti-inflammatory cytokines [1]. Developing in the bone marrow from the same myeloid progenitor as granulocytes, monocytes are eventually released into the blood where they, in humans, constitute approximately 10% of all leucocytes [2]. After a relatively short period as circulating, non-dividing cells, they either undergo apoptosis or migrate into tissues. Here, they typically differentiate into more long-lived tissue macrophages but also serve as progenitors for other cell types including dendritic cells and osteoclasts [3, 4].

Monocytes have long been considered to be a heterogeneous group of cells both in terms of morphology and function [2–5]. In humans, three distinct subsets have been described based on their differential expression of the cell surface co-receptor for bacterial lipopolysaccharide, LPS (CD14), and the low-affinity Fcγ-III receptor (CD16) [6]. Monocytes characterized by being strongly CD14 positive but lacking CD16 (CD14^high^CD16^−^) represent the great majority (90–95%) of all monocytes and are referred to as ‘classical’ monocytes. The remaining, ‘non-classical’ monocytes are CD16^+^ and have been further divided into two subsets based on their level of CD14 expression, CD14^dim^CD16^+^ and CD14^high^CD16^+^. These two minor subpopulations of cells are thought to represent more mature macrophage-like monocytes [7] and have been shown to expand during inflammatory conditions such as sepsis [8]. In addition to CD14 and CD16, a number of other cell surface molecules, including several chemokine receptors and adhesion molecules, have been shown to be differentially expressed by monocytes and are likely to reflect differences in function and migratory capacity [9, 10].

A hallmark property of monocytes is their ability to rapidly produce and secrete a broad range of cytokines in response to stimulation [11]. These include both cytokines that promote inflammation and/or cell migration (e.g. IL-1β, IL-6, TNF-α, IL-8 and MIP-1β) and such that mainly serve to dampen the inflammatory response (e.g. IL-10, TGF-β and IL-1ra). This apparent functional dichotomy has usually been explained by the same cell being capable of secreting both types of cytokines but with different kinetics and regulated by environmental conditions [12]. Attempts to relate cytokine production with the cell surface phenotype have also been made, and CD16^+^ monocytes have been reported to produce quantitatively more TNF-α [13–15] than classical monocytes while CD14^high^CD16^+^ cells have been identified as the main producers of IL-10 [16].

In an earlier study, we used the ELISpot technique to investigate LPS-induced cytokine secretion at the single-cell level and found a consistent variation in the frequencies of monocytes secreting different cytokines [17]. Here, we have employed a variant of this technique that uses fluorescent detection (FluoroSpot) allowing for detection and quantification of cells secreting two cytokines [18]. By this approach, we could demonstrate several subpopulations of monocytes with distinct cytokine-secreting profiles. These profiles were similar independent of whether cells were stimulated through Toll-like receptor (TLR)-2 or TLR-4.

## Materials and methods

### Reagents

RPMI1640, penicillin/streptomycin, HEPES and low-endotoxin (<1 EU/ml) FCS were all purchased from Invitrogen Life Technologies (Carlsbad, CA, USA). Ficoll-Paque™ PLUS was obtained from GE Healthcare Life-Sciences (Uppsala, Sweden). The RosetteSep® Monocyte enrichment cocktail was purchased from Stemcell Technologies (Grenoble, France). Ligands for TLR-2 [purified lipoteichoic acid (LTA) from *S. aureus*] and TLR-4 [ultrapure lipopolysaccharide (LPS) from *E. coli K12*] were all from InvivoGen (San Diego, CA, USA). Red blood cell (RBC) lysis buffer was purchased from BioLegend (San Diego, CA, USA). Anti-cytokine monoclonal antibodies (mAb) for ELISpot and FluoroSpot were obtained from Mabtech (Nacka Strand, Sweden) for the detection of the following cytokines: IL-1β, IL-6, TNF-α, MIP-1β, GM-CSF, IL-10 and IL-12p40. Based on their reactivity with the p40 chain common to both IL-12 and IL-23, the IL-12p40 reagents also detected IL-23-secreting cells. Streptavidin-alkaline phosphatase (SA-ALP) and BCIP/NBT (5-bromo-4-chloro-3-indolyl phosphate/nitro-blue tetrazolium) substrate were both from Mabtech as were anti-FITC-Green, Streptavidin-Red and fluorescence enhancer. The CD16^+^ monocyte isolation kit was from Miltenyi Biotec (Bergisch Gladbach, Germany). For flow cytometry, Phycoerythrin (PE)-conjugated anti-CD3 (clone HIT3a), anti-CD19 (clone HIB19), anti-CD56 (clone MEM-188) and Alexa Fluor 488-conjugated anti-CD16 (clone 3G8) mAb (including recommended isotype controls) were purchased from BioLegend. PE-conjugated anti-CD14 (clone M5E2) mAb and the recommended isotype control were from BD Biosciences (Franklin Lakes, NJ, USA) as were the BD Vacutainer® blood collection tubes containing sodium citrate.

### Monocyte enrichment using negative selection (RosetteSep®)

Blood was obtained from healthy volunteers after informed consent and with approval from the ethics committee at the Karolinska Institute, Stockholm, Sweden. The blood was collected in sodium citrate, and monocytes were enriched according to the manufacturer’s instructions using RosetteSep® (Monocyte enrichment cocktail). Briefly, 750 μl of RosetteSep cocktail was mixed with 15 ml of EDTA-treated whole blood and incubated for 20 min at room temperature (RT). The sample was then diluted 1:1 in PBS containing 2% FCS with 1 mm EDTA (PBS/FCS/EDTA) and layered on top of 15 ml Ficoll-Paque™ PLUS. After centrifugation at 1200 ***g*** for 20 min, the enriched monocytes were collected, washed twice in PBS/FCS/EDTA and suspended in cell culture medium (RPMI 1640 supplemented with 10% heat-inactivated FCS, 1 mm glutamine, 100 units/ml penicillin, 100 μg/ml streptomycin and 0.5 mm HEPES). The enriched monocytes, comprising the whole population of classical (CD14^high^CD16^−^), non-classical (CD14^dim^CD16^+^) and intermediate (CD14^high^CD16^+^) monocytes, were then counted and analysed for viability (>94% in all individuals) using the Guava ViaCount assay and the EasyCyte Mini System (Guava Technologies, Hayward, CA, USA). With this protocol, the average proportion of CD14-positive cells was 79% (*n* = 6, range 71–90%) including an average of 9% CD16^+^ monocytes. The level of contaminating T cells, B cells and NK cells in these monocyte preparations was found to be below 2% as assessed by flow cytometry.

### CD16^+^ monocyte isolation

For one set of FluoroSpot experiments, CD16^+^ monocytes were isolated using magnetic beads. For this purpose, one volume of blood was mixed with one volume of PBS and layered on top of Ficoll-Paque™ PLUS. After centrifugation at 400 ***g*** for 30 min at 22 °C, the peripheral blood mononuclear cells (PBMC) fraction was collected, washed twice and suspended in cell culture medium. Following depletion of granulocytes and NK cells, the CD16^+^ monocytes were then isolated using anti-human CD16 microbeads according to the manufacturer’s instructions (CD16^+^ monocyte isolation kit; Miltenyi Biotec). The average purity of the CD16^+^ monocyte preparations was 88% as assessed by flow cytometry.

### ELISpot/FluoroSpot assays

Low-fluorescent 96-well PVDF membrane plates (Millipore, Bedford, MA, USA) were prewet with 20 μl 35% ethanol/well for 1 min and washed five times with 200 μl sterile H_2_O. Capture anti-cytokine antibodies were diluted with sterile PBS to 15 μg/ml, and 100 μl was added to each well. After incubation overnight at +4 °C, the coated wells were washed five times with 200 μl/well of sterile PBS followed by blocking of the membrane for 30 min with 200 μl/well of cell culture medium. The medium was then removed, and 50 μl/well of the same culture medium with or without stimuli (LTA 1000 ng/ml or LPS 100 ng/ml) was added followed by the addition of 50 μl/well of cells (1000 or 3000 cells/well). Each sample was analysed in quadruplicates or triplicates. The plates were thereafter transferred to a 5%-CO_2_ incubator and incubated for 20 h at 37 °C. After incubation, cells were removed by washing five times with 200 μl/well of PBS using an automated ELISA washer (Bio-Tek Instruments Inc., Winooski, VT, USA). Detection antibodies conjugated with biotin or FITC were diluted with PBS with 0.5% FCS (PBS/FCS) to 1 μg/ml, and 100 μl was added to each well. After incubation for 2 h at RT, plates were washed as mentioned previously and wells with biotinylated detection antibodies were incubated for 1 h at RT with 100 μl/well of Streptavidin (SA) conjugated either with alkaline phosphatase (ALP) diluted 1:1000 in PBS/FCS for ELISpot or with red fluorophore, diluted to 0.5 μg/ml in PBS/FCS for FluoroSpot. At the same time, wells with FITC-labelled detection antibodies were incubated with anti-FITC-Green mAb (0.5 μg/ml in PBS/FCS). After incubation for 1 h at RT, plates were again washed five times with 200 μl/well of PBS. At this stage, the plastic underdrain of the FluoroSpot plates was removed (not for ELISpot) and the plates were incubated with either 100 μl/well of the substrate BCIP/NBT (ELISpot) or fluorescence enhancer (FluoroSpot). In ELISpot, the substrate reaction was stopped after 10 min by extensive washing in tap water and the plates were left to dry at RT. In FluoroSpot, the fluorescence enhancer was discarded after 10 min and plates were left to dry protected from light. Analysis and counting of spots were made in an ELISpot/FluoroSpot reader system (iSpot, AID, Strassberg, Germany) where fluorescent spots were counted utilizing separate filters for FITC and Cy3. Double-secreting cells were determined as spots having the same position (centre point) in an image overlay of FITC and Cy3 images (FITC+Cy3).

### Flow cytometry

Prior to examining the purity of our isolated monocytes, erythrocytes were lysed according to the manufacturer’s recommendations using RBC lysis buffer and suspended in cold PBS with 0.02% NaN_3_ and 0.5% FCS (FACS buffer). Cells were then stained for expression of CD3, CD14, CD19, CD56 and CD16 by incubating 4 × 10^5^ cells with anti-CD3-PE (10 μl), anti-CD14-PE (20 μl), anti-CD19-PE (20 μl), anti-CD56-PE (10 μl) and anti-CD16-Alexa488 (5 μl) for 15 min at 4°C in a total volume of 50 μl. After incubation, cells were washed twice in FACS buffer and analysed by flow cytometry in a Guava EasyCyte Mini System. Matched isotype controls were used to establish the background level of non-specific staining. Guava ExpressPro software (Guava Technologies) was used for data acquisition and analysis.

### Statistical analysis

Data are presented as boxplots or as means ± range. The number of individuals assessed is indicated in each figure legend. Statistical analysis was performed by applying the Wilcoxon signed-rank test using spss 16.0 software (Armonk, NY, USA). Differences were considered significant for *P < 0.05.

## Results

### Comparison of ELISpot and FluoroSpot

We have previously investigated monocyte-derived cytokine secretion in ELISpot using PBMC as the source of cells [17]. In this study, cytokine secretion was analysed with the FluoroSpot technique, and, to minimize the influence from other immune cells, experiments were performed using enriched monocytes. Purification of the monocytes was made by negative selection (RosetteSep) as pilot experiments using positive selection with anti-CD14 magnetic beads gave attenuated responses against LPS, probably as an effect of interference with the CD14 molecule (data not shown). This way, an ‘untouched’ population of enriched monocytes, comprising all monocyte subsets, was obtained.

**Figure 1:**
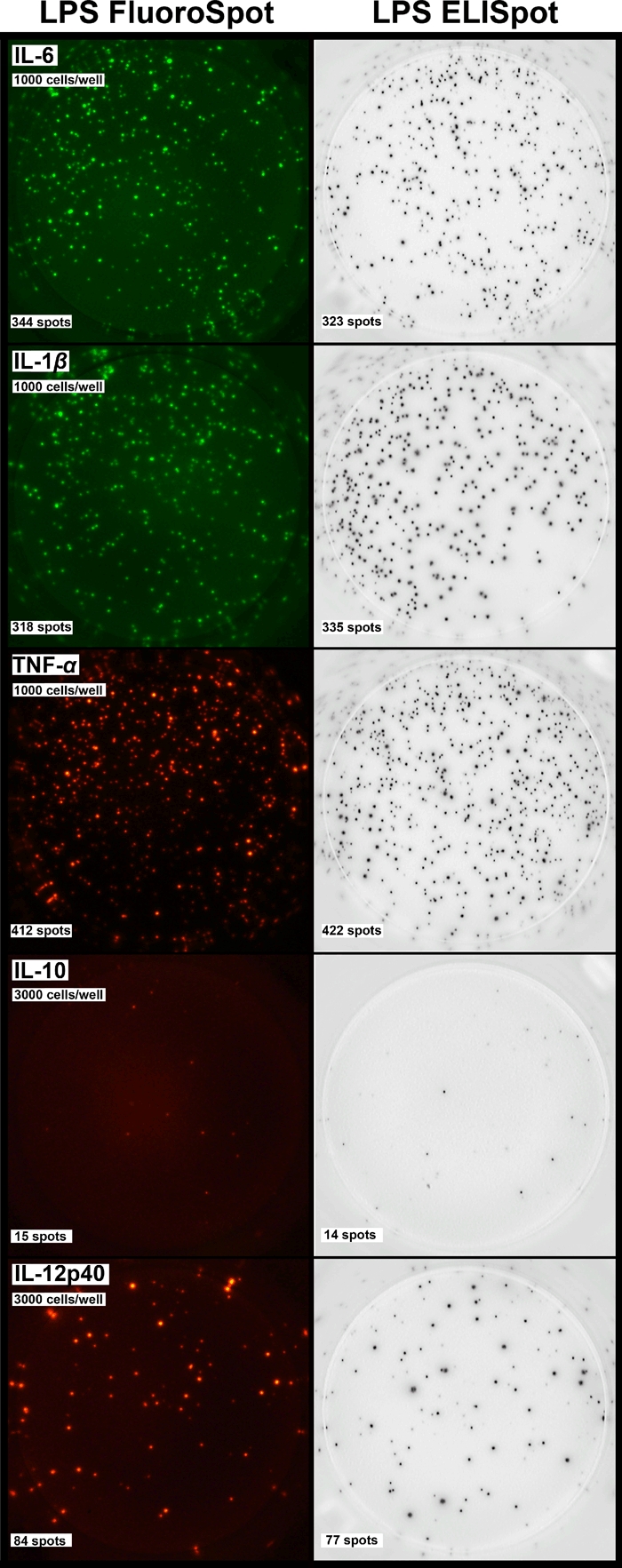
Comparison between FluoroSpot and ELISpot. Equal numbers of enriched monocytes (1000 or 3000 cells/well) were incubated for 20 h in the presence of lipopolysaccharide (50 ng/ml). The number of cells secreting IL-6, IL-1β, TNF-α, IL-10 and IL-12p40 was determined by the respective method. Similar results were obtained in two separate experiments.

To assure that the FluoroSpot assay was comparable in performance and sensitivity to the ELISpot method, enriched monocytes (1000 or 3000 cells per well) were incubated for 20 h with or without LPS (50 ng/ml) and the frequencies of cytokine-secreting cells were determined using the two methods in parallel. In the FluoroSpot assay, detection was performed either using FITC-labelled detection antibody (IL-1β, IL-6) in combination with anti-FITC-Green or with biotinylated detection antibody (TNF-α, MIP-1β, GM-CSF, IL-10 and IL-12p40) in combination with Streptavidin-Red. Both assays were performed in low-fluorescent PVDF membrane plates to allow optimal detection of fluorescent spots. As shown in Fig. 1, for a selection of the analysed cytokines, the sensitivity was comparable in the two assays. This was found to be the case for all cytokines tested (data not shown). For IL-1β and IL-6, detection was performed with both anti-FITC-Green and Streptavidin-Red, with similar results (data not shown).

### Frequencies of cytokine-secreting monocytes

**Figure 2:**
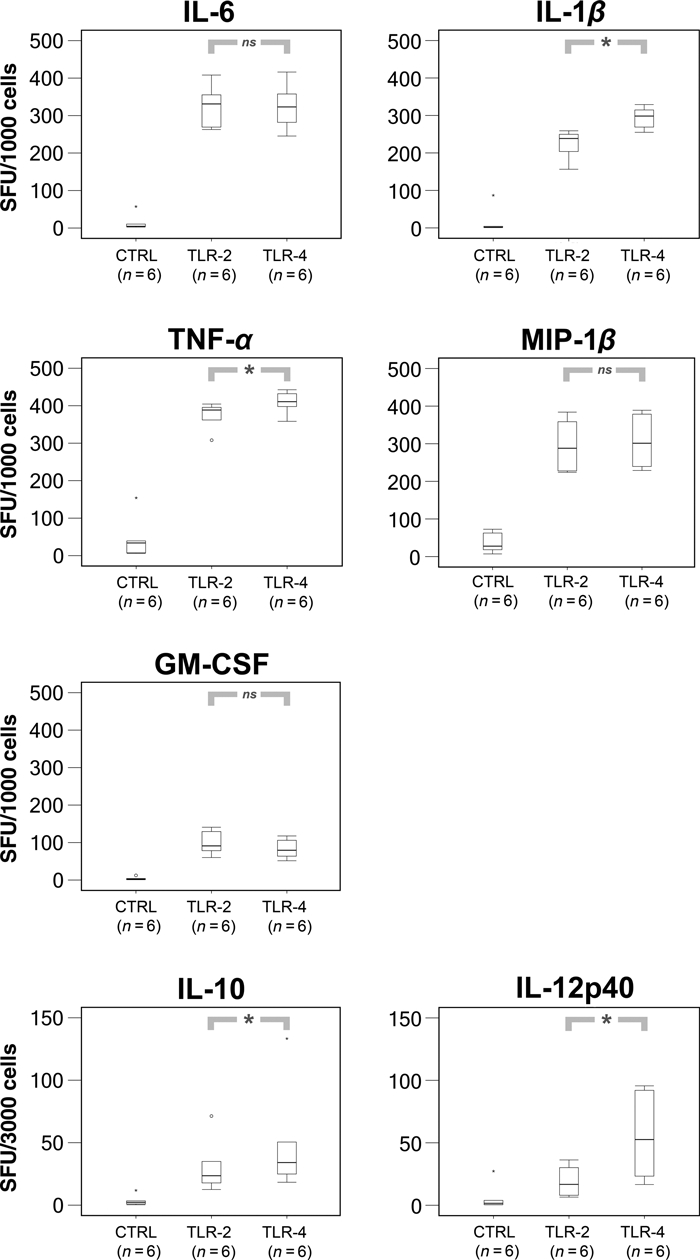
FluoroSpot analysis of cytokine secretion in response to toll-like receptor (TLR)2 or TLR4 stimulation. Enriched monocytes were incubated for 20 h with or without lipoteichoic acid (500 ng/ml) or lipopolysaccharide (50 ng/ml). The frequencies of secreting monocytes were determined using 1000 cells/well (IL-6, IL-1β, TNF-α, MIP-1β and GM-CSF) or 3000 cells/well (IL-10 and IL-12p40). Each sample and cytokine was run in quadruplicates. The number of spot-forming units (SFU) was determined in a FluoroSpot reader. Boxplots represent minimum, first quartile, median, third quartile and maximum. Differences were considered significant for *P < 0.05. TLR2 and TLR4 stimulated groups were in all cases significantly (*) upregulated as compared to negative controls. *N*, the number of individuals tested.

To investigate the numbers of cytokine-secreting cells after TLR-2 and TLR-4 stimulation, enriched monocytes from six healthy donors were incubated for 20 h in the absence or presence of LTA (500 ng/ml) or LPS (50 ng/ml) and analysed in FluoroSpot for the secretion of seven monocyte-derived cytokines (Fig. 2). As previously observed, the number of secreting monocytes varied depending on which cytokine was investigated and, to a certain extent, also on the type of TLR ligand to which the cells were exposed. Based on the average numbers of responding cells, monocytes could be divided into three subgroups where IL-1β, IL-6, TNF-α and MIP-1β were secreted by larger populations of responding cells (25.9–39.2%) compared with the smaller populations of GM-CSF (9.1%), IL-10 (1.3%) and IL-12p40 (1.2%).

### FluoroSpot analysis of IL-6- and IL-1β-secreting monocytes co-secreting TNF-α and MIP-1β

After having established the number of secreting cells for each cytokine and stimulus, we went on to investigate co-secretion among the most frequent cytokines secreted, that is IL-1β, IL-6, TNF-α and MIP-1β. For this purpose, plates were coated with anti-IL-1β or anti-IL-6 antibodies in combination with antibodies to either TNF-α or MIP-1β, and detection was performed using secondary reagents coupled with green fluorophore (IL-1β and IL-6) or red fluorophore (TNF-α, MIP-1β and IL-1β). By this approach, three distinct subpopulations could potentially be revealed: FITC – single-secreting cells, FITC + Cy3 – double-secreting cells and Cy3 – single-secreting cells.

**Figure 3:**
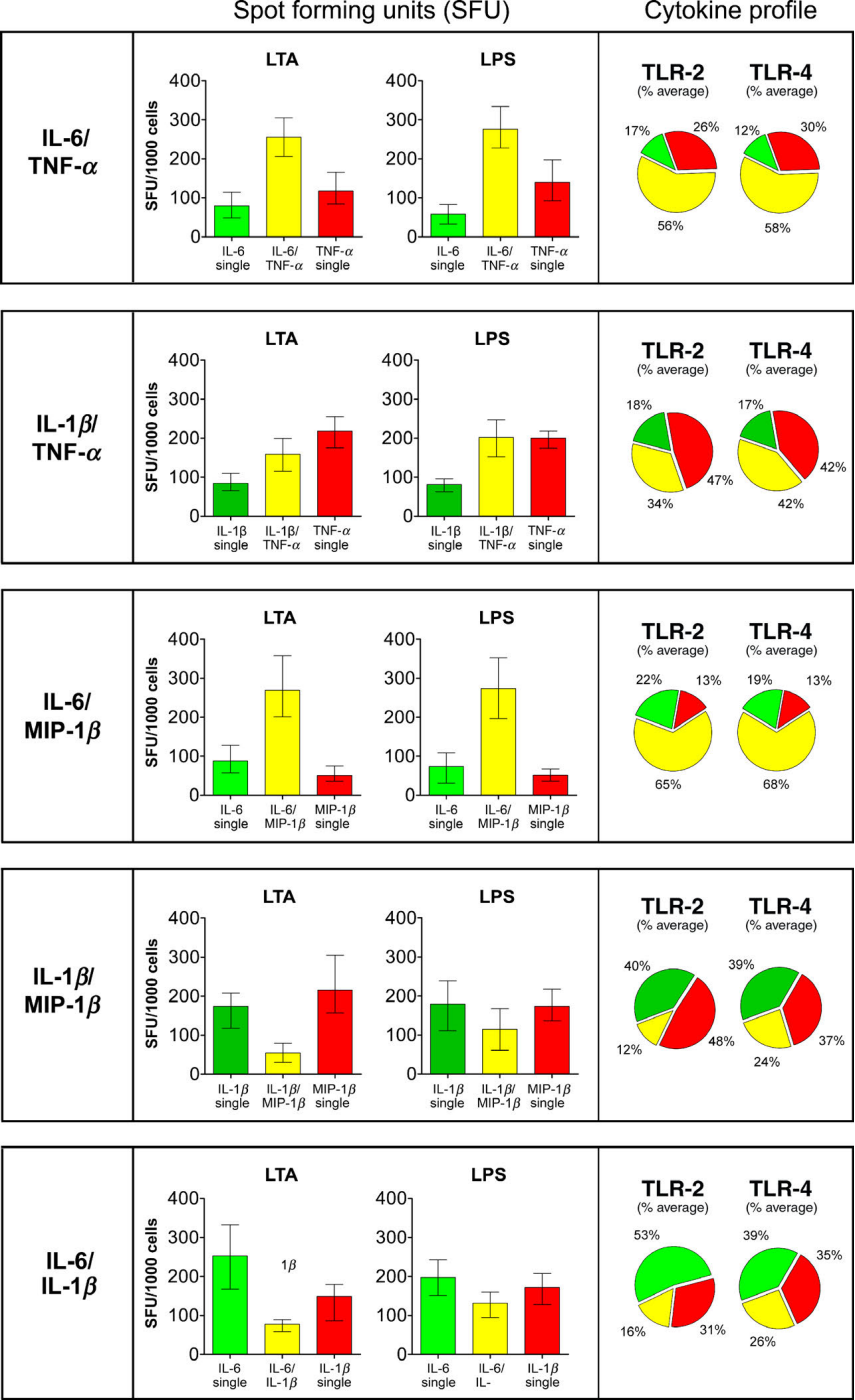
FluoroSpot analysis of IL-6- and IL-1β-secreting monocytes co-secreting TNF-α or MIP-1β. Enriched monocytes (1000 cells/well) were incubated for 20h and analysed for the co-secretion of IL-6/TNF-α, IL-1β/TNF-α, IL-6/MIP-1β, IL-1β/MIP-1β and IL-6/IL-1β in response to lipoteichoic acid (500 ng/ml) or lipopolysaccharide (50 ng/ml). Analysis of each cytokine combination revealed three distinct populations: monocytes secreting either one of the cytokines (green or red bars) or monocytes secreting both cytokines (yellow bars). Values represent the mean ± range of six individuals (*n* = 6). Pie charts show the average percentage of toll-like receptor-activated monocytes secreting either one of the cytokines or both.

As shown in Fig. 3, there was a substantial overlap in secretion between all four cytokines. For instance, of the monocytes secreting IL-6, more than 75% concomitantly secreted TNF-α. A similar relationship was observed in the IL-1β population where the overlap with TNF-α was 65% and 71% for LTA and LPS, respectively. In contrast, when IL-6 or IL-1β was combined with MIP-1β, a different co-secreting relationship was observed (Fig. 3). Thus, while a majority of IL-6-secreting monocytes also secreted MIP-1β, the IL-1β monocytes displayed a much more restricted percentage of co-secretion. Similarly, when IL-1β (Streptavidin-Red) and IL-6 (anti-FITC-Green) were combined, less than half of the IL-1β population was found to co-secrete IL-6 (Fig. 3).

### FluoroSpot analysis of IL-6- and IL-1β-secreting monocytes co-secreting GM-CSF

**Figure 4:**
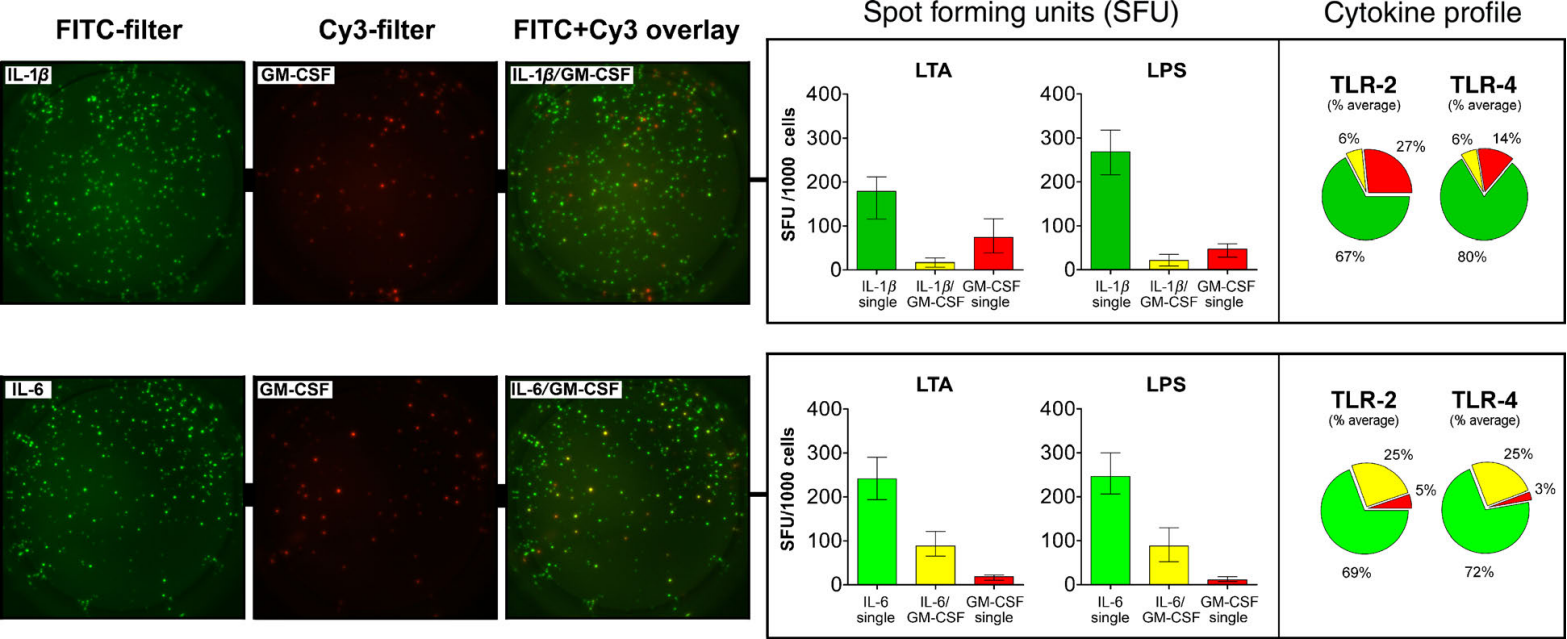
FluoroSpot analysis of IL-6- and IL-1β-secreting monocytes co-secreting GM-CSF. Enriched monocytes (1000 cells/well) were incubated for 20 h in FluoroSpot plates and analysed for the co-secretion of IL-1β/GM-CSF and IL-6/GM-CSF in response to lipoteichoic acid (500 ng/ml) or lipopolysaccharide (50 ng/ml). Analysis of each cytokine combination revealed three distinct populations: monocytes secreting either one of the cytokines (green or red bars) or monocytes secreting both cytokines (yellow bars). Values represent the mean ± range of six individuals (*n* = 6). Pie charts show the average percentage of toll-like receptor-activated monocytes secreting either one of the cytokines or both. In the left panel, representative examples of the FluoroSpot results are shown for IL-1β/GM-CSF and IL-6/GM-CSF.

GM-CSF-secreting monocytes, when combined with IL-1β, displayed a limited degree of co-secretion (Fig. 4). Consequently, of the total number of monocytes secreting GM-CSF and/or IL-1β in response to stimulation, only 6% secreted both cytokines simultaneously. In contrast, the great majority of GM-CSF-positive cells also secreted IL-6 (83% in response to LTA, 89% with LPS).

### FluoroSpot analysis of IL-6- and IL-1β-secreting monocytes co-secreting IL-10 and IL-12p40

**Figure 5:**
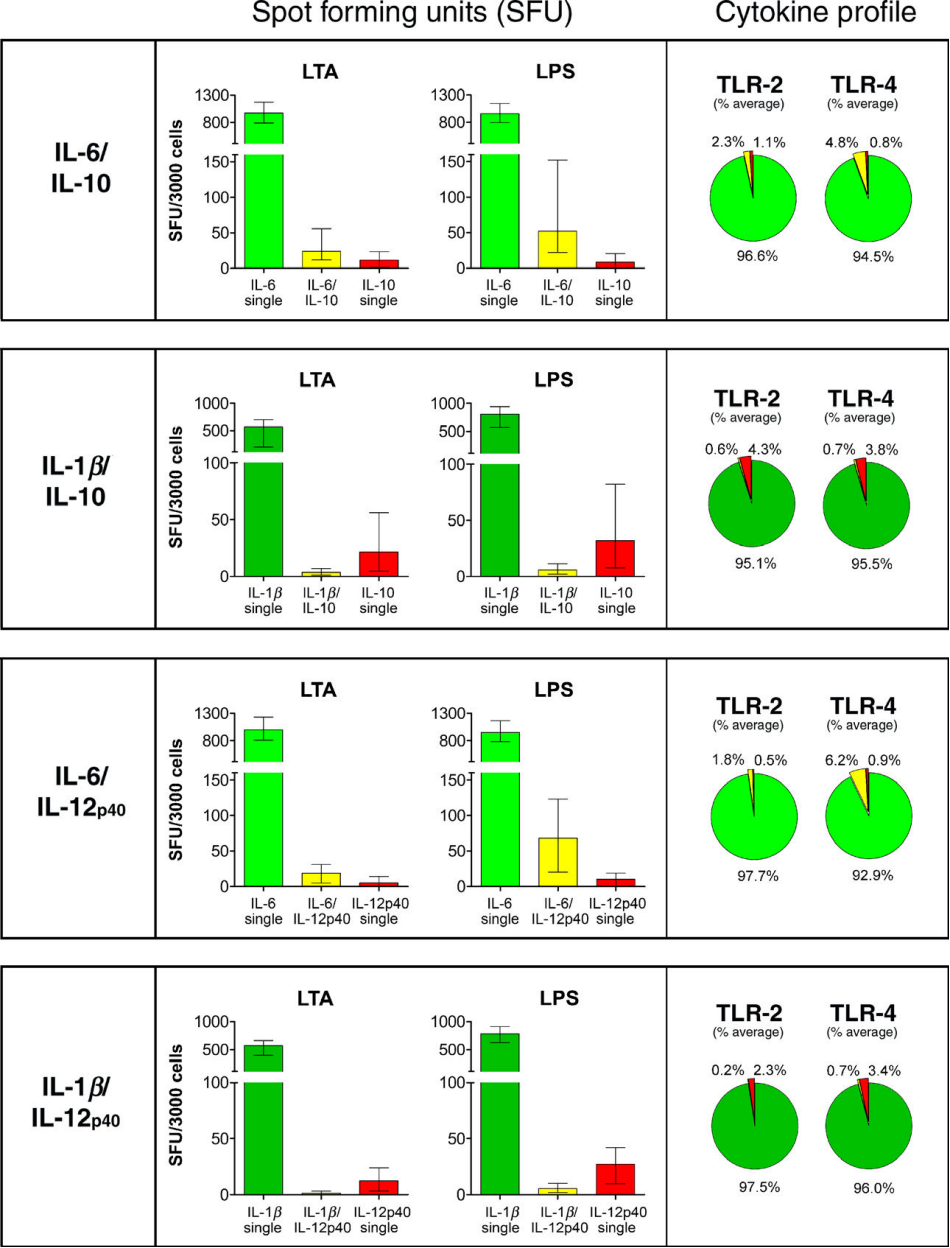
FluoroSpot analysis of IL-6and IL-1β-secreting monocytes co-secreting IL-10 or IL-12p40. Enriched monocytes (3000 cells/well) were incubated for 20 h in FluoroSpot plates and analysed for the co-secretion of IL-6/IL-10, IL-1β/IL-10, IL-6/IL-12p40 and IL-1β/IL-12p40 in response to lipoteichoic acid (500 ng/ml) or lipopolysaccharide (50 ng/ml). Analysis of each cytokine combination revealed three distinct populations: monocytes secreting either one of the cytokines (green or red bars) or monocytes secreting both cytokines (yellow bars). Values represent the mean ± range of six individuals (*n* = 6). Pie charts show the average percentage of toll-like receptor-activated monocytes secreting either one of the cytokines or both.

Compared with the high frequencies of monocytes secreting IL-1β, IL-6, TNF-α and MIP-1β, (25.9–39.2%) only a minority secreted IL-10 and IL-12p40 (<2%). Lacking suitable reagents for the double staining, we could not establish to what extent IL-10 and IL-12p40 were produced by the same or different populations of cells. However, simultaneous staining for IL-12p40 and IL-6 showed that a majority of the IL-12p40-secreting monocytes were also positive for IL-6 (78% with LTA and 87% with LPS), and a similar but slightly lower overlap was observed for IL-10 and IL-6 (Fig. 5). In contrast, only a small proportion of the monocytes secreting IL-10 and/or IL-12p40 showed a simultaneous secretion of IL-1β (<17%).

### CD16^+^ monocytes

Although CD16^+^ monocytes are recognized as key producers of TNF-α, their production of other cytokines is less clear, and contradicting data exist as to their secretion of IL-10 [13–16]. To evaluate their cytokine profile, CD16^+^ non-classical and intermediate monocytes (CD14^dim^CD16^+^ and CD14^high^CD16^+^) were separated from PBMC using anti-CD16 magnetic beads and analysed in FluoroSpot in the absence or presence of LPS (1000 cells per well).

**Figure 6:**
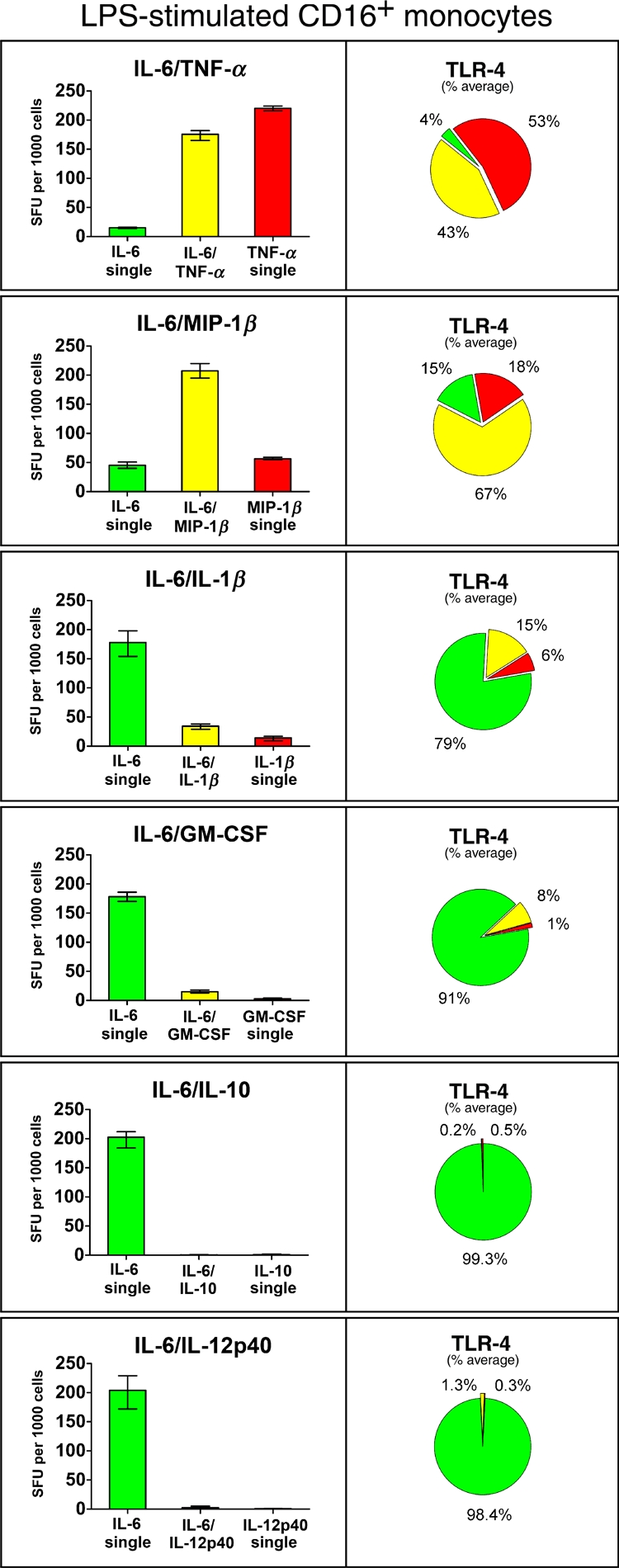
FluoroSpot analysis of cytokine co-secretion by lipopolysaccharide (LPS)-stimulated CD16^+^ monocytes. Purified CD16^+^ monocytes (1000 cells/well) were incubated for 20 h in FluoroSpot plates and analysed for the co-secretion of IL-6/TNF-α, IL-6/MIP-1β, IL-6/IL-1β, IL-6/GM-CSF and IL-6/IL-10, IL-6/IL-12p40 in response to LPS. Values represent the mean ± range of triplicates. Pie charts show the average percentage of LPS-activated CD16^+^ monocytes secreting either one of the cytokines or both. Monocytes cultured in medium alone are not shown but contained few cytokine-secreting cells (<52 spots for TNF-α, <22 spots for IL-6, <14 spots for MIP-1β, <3 spots for IL-1β, GM-CSF, IL-10 and IL-12p40). Similar results were obtained in two separate experiments.

As expected, a large number of the CD16^+^ monocytes secreted TNF-α and of this population, approximately half co-secreted IL-6 (Fig. 6). MIP-1β was secreted by a similar number of cells as IL-6, and they also shared a high degree of co-secretion. In contrast, only a small percentage of the cells secreted IL-1β or GM-CSF and very few cells secreted IL-10 or IL-12p40 (Fig. 6).

## Discussion

Monocytes, macrophages and dendritic cells are key effector cells of innate immunity and collectively make up the mononuclear phagocyte system (MPS) [19]. Apart from their fundamental role in host defence against invading micro-organisms, these cells are also critical in maintaining various aspects of tissue homeostasis and take part in a variety of regulatory functions, from the clearance of apoptotic cells and toxic compounds to the bridging of the adaptive immune response [1, 20]. Within this network, circulating monocytes have traditionally been regarded as a large pool of precursor cells, which when needed, can extravasate and differentiate into macrophages and dendritic cells with a remarkable diversity in phenotype [3, 12]. Because of this cellular plasticity, monocyte heterogeneity has naturally been suggested and has been an active area of research especially since the discovery of classical (CD16 negative) and non-classical (CD16 positive) monocyte subpopulations [21]. Although this classification has been useful in delineating several aspects of monocyte biology, it has at the same time been technically difficult to associate the expression of CD16 to that of cellular functionality in terms of cytokine production.

For example, cytokine determinations based on ELISA and RT-PCR have only resulted in crude characterizations as neither assay permits resolution at the cellular level [13–16]. Furthermore, intracellular staining in combination with flow cytometry may not provide an accurate picture of what is actually secreted by the monocytes and could be complicated by the technical limitations of protein transport inhibitors (e.g. Brefeldin A) required for protein accumulation and optimal staining [22]. Thus, to further extend our knowledge into the functional properties of monocytes, we decided in the present study to approach the issue of cytokines using the newly developed FluoroSpot technique. This method measures the simultaneous secretion of two cytokines at the single-cell level and can be performed using only a few hundred monocytes per well, offering a clear advantage over alternative methods such as flow cytometry and ELISA.

By this approach, we were able to determine the numbers of monocytes secreting IL-1β, IL-6, TNF-α, MIP-1β, GM-CSF, IL-10 and IL-12p40 in response to stimulation. Of these, the first four were secreted by larger populations of the cells (25.9–39.2%), whereas GM-CSF was produced by a smaller population (9.1%) and IL-10 and IL-12p40 only by a few per cent (1.2–1.3%) of the monocytes. This pattern of secretion was surprisingly consistent between different donors and, apart from IL-1β and IL-12p40, very similar for both LTA and LPS stimulation (Fig. 2).

Given the similar and high numbers of monocytes secreting IL-1β, IL-6, TNF-α and MIP-1β, it was natural to assume that these cytokines were, in fact, all secreted by the same population of cells. However, once we were able to combine these cytokines in two-colour FluoroSpot, the results revealed a much more intricate, but consistent pattern of co-secretion. Thus, while the two cytokines IL-6 and MIP-1β showed a high degree of secretory overlap, co-secretion of IL-1β and MIP-1β was only observed in a minority of the cells (Fig. 3). This low frequency of double-secreting cells was also observed when combining IL-1β with IL-6, whereas, in contrast, TNF-α-secreting monocytes shared a high degree of co-secretion with both (Fig. 3). These results clearly show that despite being properly activated, individual monocytes respond differently after TLR stimulation and appear to carry an inbuilt capacity to secrete a certain set of cytokines. Similarly, when further investigating co-secretion of GM-CSF, the great majority of these cells (approximately 90%) also secreted IL-6, whereas only about 30% showed a simultaneous secretion of IL-1β (Fig. 4). This somewhat dichotomous relationship between IL-6 and IL-1β was even more evident when the co-secretion of IL-10 and IL-12p40 was considered (Fig. 5).

While our results provide further support for the heterogeneous nature of monocytes, they also demonstrate the possibility of dividing these cells into several subsets based on differences in cytokine-secreting capacity. A similar division of T cells into subpopulations, each with their own unique cytokine profile, has proven very useful and has allowed for the characterization of a number of T cell subsets with distinct functional properties [23]. Although cytokine production by monocytes may be more susceptible to environmental manipulation than what is the case for T cells, it is tempting to hypothesize that the analogous identification of monocyte subpopulations may be of similar importance when trying to better understand the role and function of these cells. In this context, it is well known that many of the regulatory effects of cytokines are executed at the local level between neighbouring cells making the cytokine-secreting profile of individual monocytes important. Furthermore, one can speculate that the cytokine profiles described here for activated monocytes are maintained when these cells extravasate into tissues, forming a functional foundation for the development of macrophages with distinct phenotypic properties.

Differences in cytokine production by monocytes have been reported previously, and CD16^+^ monocytes have been claimed to be the major source of TNF-α [13–15]. In line with this, we could indeed observe an increased number of TNF-α-secreting cells in the CD16^+^ population when compared to the number of cells secreting IL-6 or MIP-1β (Fig. 6). However, as the CD16^+^ subset only constitute a small fraction of all monocytes, this subpopulation can, in practice, only account for a minority of all TNF-α-secreting cells. This was also confirmed when CD16-depleted cells where analysed in FluoroSpot as depletion had a marginal effect on the number of cells secreting TNF-α (data not shown). Furthermore, it has been claimed that the monocytes secreting IL-10 are primarily found in the CD14^high^CD16^+^ subpopulation [16] and that this subpopulation thus might serve as an anti-inflammatory counterpart to the inflammatory profile of the CD14^dim^CD16^+^ subset. However, in line with previous investigators [13–15], we were not able to confirm these results as very few IL-10-secreting cells were found within the CD16^+^ population (Fig. 6).

Finally, while the results of this study add a further level of complexity to monocytes, we believe that they also provide a meaningful basis for further characterization of these cells from a functional perspective and may therefore serve as an important complement to the currently accepted subclassification of monocytes. We also think that the same approach may be used to study cytokine secretion by other members of the MPS, including macrophages, dendritic cells and bone marrow–derived precursors and that such characterization may offer new insights into their functional/developmental relationships.
